# A systematic review of brachial plexus injuries after caesarean birth: challenging delivery?

**DOI:** 10.1186/s12884-023-05696-1

**Published:** 2023-05-17

**Authors:** Shireen Jaufuraully, Anjana Lakshmi Narasimhan, Daniel Stott, George Attilakos, Dimitrios Siassakos

**Affiliations:** 1 Elizabeth Garrett Anderson Institute for Women’s Health, University College London, London, UK; 2 Wellcome / EPSRC Centre for Interventional and Surgical Sciences, University College London, London, UK; 3 UCL Medical School, University College London, London, UK; 4 Elizabeth Garrett Anderson Wing, University College Hospital, London, UK; 5 National Institute for Health Research (NIHR) University College London Hospitals Biomedical Research Centre (BRC), London, UK

**Keywords:** Birth injury, Brachial plexus injury, Caesarean section, erb’s palsy, Neonatal injury, Operative birth, Zavanelli

## Abstract

**Background:**

Caesarean section (CS) is widely perceived as protective against obstetric brachial plexus injury (BPI), but few studies acknowledge the factors associated with such injury. The objectives of this study were therefore to aggregate cases of BPI after CS, and to illuminate risk factors for BPI.

**Methods:**

Pubmed Central, EMBASE and MEDLINE databases were searched using free text: (“brachial plexus injury” or “brachial plexus injuries” or “brachial plexus palsy” or “brachial plexus palsies” or “Erb’s palsy” or “Erb’s palsies” or “brachial plexus birth injury” or “brachial plexus birth palsy”) and (“caesarean” or “cesarean” or “Zavanelli” or “cesarian” or “caesarian” or “shoulder dystocia”). Studies with clinical details of BPI after CS were included. Studies were assessed using the National Institutes for Healthy Study Quality Assessment Tool for Case Series, Cohort and Case-Control Studies.

**Main results:**

39 studies were eligible. 299 infants sustained BPI after CS. 53% of cases with BPI after CS had risk factors for likely challenging handling/manipulation of the fetus prior to delivery, in the presence of considerable maternal or fetal concerns, and/or in the presence of poor access due to obesity or adhesions.

**Conclusions:**

In the presence of factors that would predispose to a challenging delivery, it is difficult to justify that BPI could occur due to in-utero, antepartum events alone. Surgeons should exercise care when operating on women with these risk factors.

**Supplementary Information:**

The online version contains supplementary material available at 10.1186/s12884-023-05696-1.

## Introduction

The brachial plexus is a group of nerves that innervate the upper limb. They consist of the 5th − 8th cervical, and the 1st thoracic anterior rami [1], known as C5-T1. Obstetric brachial plexus injury (BPI) has been attributed to excessive lateral traction by the accoucheur [2]. It affects between 0.15 and 3 per 1000 live births, depending on the country of birth [3]. Injury to the brachial plexus can be debilitating [4]. Children with more severe injuries have been noted to have behavioural and developmental difficulties [2]. Compared with the general population, parents of children with BPI have a lower quality of life [5] and higher stress levels [6].

BPI not only has a devastating impact on the lives of babies and their parents, but is a common source of litigation globally. Between 2000 and 2010, £103 million was spent on litigation costs associated with obstetric BPI and shoulder dystocia in the UK alone [7]. Clearly prevention is important. Caesarean section (CS) is protective against BPIs and should be considered for mothers of high-risk babies [6, 8]. Even though rates of CS are increasing globally [9], the incidence of BPI has been static for the last 50 years [10], and even increasing in recent years [11]. Moreover, prediction is imprecise [12], and only 60% of obstetric BPIs occur after documented shoulder dystocia [13]. Alternative theories have been developed to explain BPI, in addition to iatrogenic injury caused by the accoucheur. The ‘in-utero insult’ theory attributes BPI to antepartum events such as malpresentation [14], the propulsive forces of labour, and uterine anomalies [15]. However, malpresentation deliveries are unlikely to be delivered without some degree of force [16]. Such theories have been met with criticism, particularly after the implementation of programmes such as PROMPT (Practical Obstetric Multi-Professional Training) [12, 17, 18], where practical training in the manoeuvres used to alleviate shoulder dystocia during vaginal birth are associated with a dramatic reduction in rates of permanent BPI to zero, which could not happen if other mechanisms can cause BPI.

This systematic review aims to explore documented cases of BPI after CS in the literature, and whether they support a theory that BPI can be caused by the intrauterine environment and not the accoucheur(s).

## Methods

### Protocol and registration

The study protocol was registered on PROSPERO international prospective register of systematic reviews (CRD42021253929). The Preferred Reporting Items for Systematic Reviews and Meta-Analyses (PRISMA) guidelines were used to conduct the systematic review.

### Eligibility criteria

All studies were included if there were any cases with clinical details of brachial plexus injury after CS. Systematic reviews, review articles and conference abstracts were excluded. There were no language restrictions.

### Search Strategy

Pubmed Central, EMBASE and MEDLINE databases were searched using free text: (“brachial plexus injury” or “brachial plexus injuries” or “brachial plexus palsy” or “brachial plexus palsies” or “Erb’s palsy” or “Erb’s palsies” or “brachial plexus birth injury” or “brachial plexus birth palsy”) and (“caesarean” or “cesarean” or “Zavanelli” or “cesarian” or “caesarian” or “shoulder dystocia”).

### Study selection

2 reviewers (ALN and SJ) independently reviewed the title and abstracts of all papers generated from the searches. Full texts of potentially relevant papers were downloaded and read independently to ascertain relevance. Disagreements were settled by consensus. A feature of EMBASE removed duplicates automatically from EMBASE and Medline. Duplicates were removed manually from Pubmed Central. Full texts were available online. Studies were excluded if there were no cases of BPI after CS, if there appeared to be the same patient cohort and results in studies published by the same authors, and if no further details other than ‘BPI after CS’ were provided.

### Data extraction

Data was independently extracted by ALN and SJ and inputted onto a Microsoft Excel for Mac (Excel Version 16.49) spreadsheet. Headings were: ‘Title’, ‘First Author’, ‘Journal’, ‘Year published’, ‘Type of Study’, ‘Number of CS out of total deliveries with BPI’, and a column to document further delivery details (supporting information).

### Quality assessment

ALN and SJ screened study quality and risk of bias together using the National Institutes for Healthy Study Quality Assessment Tool for Case Series, Cohort and Case-Control Studies.

### Result synthesis

Meta-analysis was not performed due to the heterogeneity of studies. Tables were created using Microsoft Excel for Mac Version 16.49.

## Results

### Study selection

**Fig. 1:**
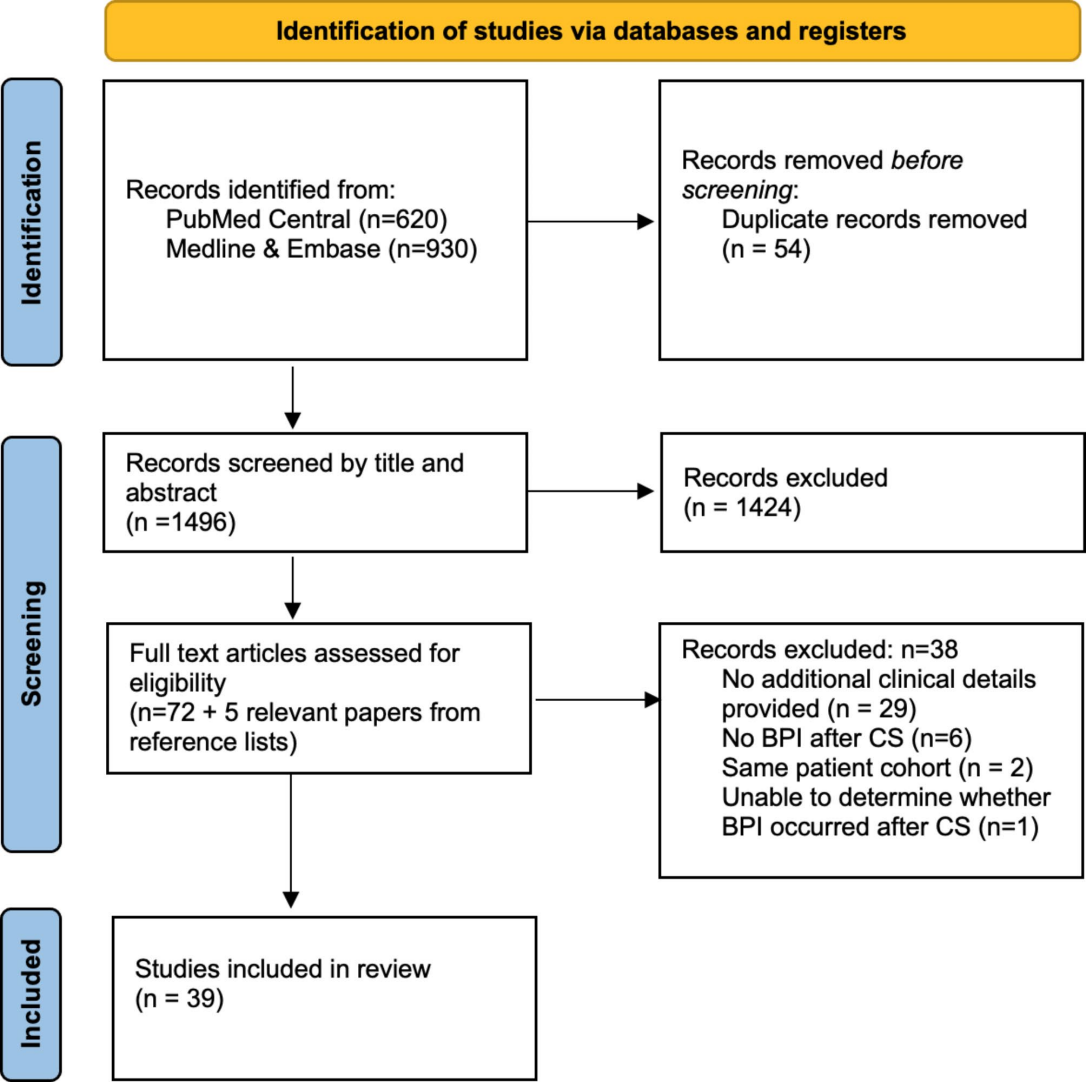
Flow diagram for study selection adapted from PRISMA 2020

The electronic searches identified 1550 papers published up to February 2022 (Fig. 1). There were 620 from Pubmed central, and 930 from Medline and Embase. 54 duplicates were removed, and the remaining 1496 studies were screened. 1424 were irrelevant and excluded, which left 72 full text articles to review. No translation tools were required. 38 studies were excluded for the following reasons: no further details on BPI after CS (n = 29), no cases of BPI after CS (n = 6), same patient cohort previously published by the same authors (n = 2), inability to ascertain whether there were any cases of BPI after CS (n = 1). Reference lists from selected papers were screened; five additional studies were found and were included. 39 studies were included in the systematic review.

### Study characteristics

There were 24 case series, 10 cohort studies, and five case controls. Study characteristics are summarised in Table 1.

**Table 1:** Study characteristics. RFs = risk factors (place after line 147)

Author	Year Published	Study design	No. of cases with BPI after CS	RFs for challenging delivery	Cases with RFs/total CS with BPI
Backe et al. [19]	2008	Case series	2	Malpresentation	2/2
Donnelly et al. [20]	2002	Case control	1	Malpresentation	1/1
Evans-Jones et al. [13]	2003	Case series	5	Malpresentation, obstructed labor	4/5
Graham et al. [14]	1997	Case series	1	Malpresentation	1/1
Gurewitsch et al. [21]	2006	Cohort	1	Malpresentation	1/1
Iffy et al. [22]	2005	Case series	2	Malpresentation, Zavanelli	2/2
Johnson et al. [23]	2020	Cohort	3	Malpresentation, emergency CS, obstructed labor	3/3
Mcfarland et al. [24]	1986	Case control	4	Malpresentation, emergency CS, failed instrumental delivery	3/4
Sherman et al. [25]	2010	Case series	16	Malpresentation	9/16
Sibiñski et al. [26]	2007	Case series	3	Malpresentation	1/3
Walsh et al. [12]	2011	Case series	5	Malpresentation, failed instrumental delivery	4/5
Wolf et al. [27]	2000	Case control	1	Malpresentation	1/1
Alexander et al. [28]	2007	Cohort	9	Obstructed labor, emergency CS	5/9
Alsubhi et al. [29]	2011	Case series	13	Obstructed labor, fetal macrosomia	Unknown
Bhat et al. [30]	1995	Case control	8	Obstructed labor	8/8
Carsi et al. [31]	2015	Case series	2	Obstructed labor, raised BMI	2/2
Gherman et al. [16]	1997	Case series	17	Obstructed labor, malpresentation	17/17
Ogbemudia et al. [32]	2012	Case series	1	Obstructed labor	1/1
Malik et al. [33]	2014	Case series	2	Obstructed labor, failed instrumental delivery	2/2
Iffy et al. [34]	2003	Case series	1	Failed instrumental delivery	1/1
Alfonso et al. [35]	2004	Case series	1	Emergency CS	1/1
Al-Qattan [36]	2016	Case series	1	Emergency CS	1/1
Fogel I et al. [37]	2021	Cohort	3	Emergency CS, fetal macrosomia	3/3
Haley et al. [38]	2019	Cohort	154	Emergency CS	50/154
Ouzounian et al. [39]	2013	Cohort	1	Emergency CS	1/1
Rehm et al. [40]	2019	Cohort	4	Emergency CS	4/4
Sinclair et al. [41]	2008	Case series	1	Emergency CS	1/1
Wang KK et al. [42]	2020	Case series	3	Emergency CS	2/3
Aberg et al. [43]	2016	Cohort	18	Fetal macrosomia	6/18
Al-Qattan [44]	1996	Case series	1	Fetal macrosomia	1/1
Ecker et al. [45]	1997	Cohort	2	Fetal macrosomia	1/2
Torki et al. [46]	2012	Case series	1	Fetal macrosomia	1/1
Eken at el [47]	2015	Case series	4	Raised BMI	4/4
Doty et al. [48]	2020	Cohort	1	Zavanelli	1/1
Gherman et al. [49]	2010	Case series	1	Zavanelli	1/1
Iffy et al. [50]	2007	Case series	2	Zavanelli	2/2
Kenaan et al. [51]	2003	Case series	2	Zavanelli	2/2
Sandmire et al. [52]	2003	Case series	1	Zavanelli	1/1
Turrentine et al. [53]	1999	Case control	1	Zavanelli	1/1
				**Total**	**152/286 = 53%**

### Quality Assessment

Most studies were of good quality, with six studies of fair quality and one study of poor quality (supporting information).

### Malpresentation & difficult extraction

Papers described BPI in 33 infants after CS for malpresentation or following difficult extraction

Nineteen babies were diagnosed with BPI after CS for breech presentation. Three babies were preterm. A singleton baby was born by emergency CS due to ‘preterm breech presentation’. The surgeon reported difficulty in extracting the shoulders through the uterine incision. This injury was transient, and the baby was born with normal Apgars [19]. Similarly, delivery of a 32-week baby weighing 1357 g was described as a ‘difficult extraction’. It made a complete recovery [27]. A baby with a weight of 830 g, of unknown gestation, also sustained BPI [24]. In a further study, a term, 3.84 kg baby was born via repeat elective CS in good condition. There was a ‘fair amount of scarring and muscle’ necessitating transection of the rectus. The baby had a left sided Erb’s palsy. The Consultant Neonatologist concluded it to be an acute injury as there were no contractures to suggest it was an in-utero insult. The baby also sustained a hairline fracture of the clavicle; indicating traumatic delivery. The injury persisted after 8 years with muscle atrophy [22]. In contrast, in one study, an infant was born without apparent difficulty and still sustained a BPI [23].

Sherman et al. reported on four babies delivered in breech presentation who sustained BPI after CS; two of these were after attempted version of the fetus, and two were due to difficulties in extracting the head in a cephalic fetus, hence undergoing breech extraction [25]. A study of litigated cases following permanent BPI described a baby born after failed external cephalic version (ECV) [21]. Gherman et al., when assessing rates of BPI after CS, excluded 9 breech babies who sustained BPIs from their analysis as they attributed it to traumatic delivery [16]. When comparing BPI rates over two time periods, Walsh et al. described a baby born by CS with compound breech and hand presentation – this injury was transient [12].

Nine caesareans were performed for transverse lie; three babies weighing approximately 2.6 kg, apparently born without difficulty, sustained BPIs [14, 19, 20]. Of note, one was an elective repeat CS [14], and one an emergency CS for a second twin born at 35 weeks’ gestation with normal Apgars (the injury was transient) [19]. Another baby was born by elective CS at 38 weeks for unstable lie. It was described as a difficult delivery due to being back down. This injury was transient [12]. There were no further details for the remaining five cases [25, 26].

Gherman et al. also excluded two babies with impacted fetal head at CS from their report of BPI after CS [16]. In other studies, there were four cases of difficult extraction of a cephalic fetus. One baby with hand presentation underwent cephalic version during emergency CS and weighed 3560g^1^ [3]. When describing one case, the operation note reported difficulty: ‘fetal head lodged deep in the pelvis and CS performed due to arrest of labour.’ The baby had evidence of nerve root avulsion at one year of age. The authors postulated that it could be due to longstanding brachial plexus stretch or antecedent factors such as ECV [16] but the difficult extraction means that injury at birth is equally if not more likely. Sherman et al. stated that iatrogenic damage during a difficult extraction is possible after describing a case of similarly difficult extraction of a cephalic baby [25].

### Obstructed labour and failure to progress

Nine papers describe BPI in 36 neonates after CS for failure to progress or obstructed labour. There were 15 cases of radial nerve palsy; 13 were CS for suspected cephalopelvic disproportion (CPD) or failure to progress. Infants weighed between 3 and 4.1 kg [29]. In a case series of transient radial nerve palsy, two infants were described. One term infant was born to a primiparous woman with known fibroids. She underwent CS due to failure to progress in the first stage. The baby weighed 3.7 kg and had a left wrist drop. The latter infant was born to a woman with a BMI of 40 and GDM. The baby was born at 38 + 2 weeks’ gestation and weighed 3.6 kg. Both arms were bruised [31].

There were multiple cases of BPI in low resource settings. Eight babies sustained Erb’s palsies after CS for obstructed labour. Bhat et al. reported that the majority did not have adequate antenatal care. All completely recovered after 6 months [30]. A further case study describes a 25 year-old primiparous woman who underwent CS for prolonged obstructed labour. The baby suffered from ipsilateral Klumpke’s palsy, a fractured left clavicle and humerus. It was cephalic and weighed 3.9 kg. The authors postulated that the force required to deliver the head in obstructed labour may be sufficient to cause fractures and nerve palsy, and concluded there was a need to increase advocacy in developing countries to ensure that primiparous women are attended by qualified obstetricians, and difficult CS are performed by experienced obstetricians [32]. A 2.5 kg neonate was also born by CS after prolonged obstructed labour in a study on traumatic neuropraxias [33].

These types of birth injury are not unique to low resource countries; there are several cases of BPI after obstructed labour reported in high resource settings too. In one study, a neonate was delivered after two hours of maternal pushing and no progress past + 1 to spines [23]. In another study, two neonates, weighing 3500 g and 3048 g sustained BPI after CS for failure to progress [13]. A further four women underwent CS for ‘dystocia’ and their babies sustained BPIs [28]. Gherman et al. describe 6 cases of BPI, considered by them non-iatrogenic [16]. These cases are summarised in Table 2; risk factors for challenging delivery are evident in every single case in our opinion.

**Table 2:** Cases of BPI after obstructed labour. Adapted from Gherman et al. [16]

Case	Age	Parity	Gestation	Clinical details	Neonatal weight(g)	Location of Erb’s palsy
1	37	1	38 + 4	Failure to progress at 9 cm. 6 × 7 cm lower uterine segment fibroid.	2850	Left sided (posterior shoulder) persistent at 29 months
2	26	0	39 + 1	Arrest of descent at spines. 4 h second stage due to operating theatre availability.	4225	Right sided (anterior shoulder) persistent at 12 months
3	25	0	40 + 2	Failure to progress at 8 cm after 13 h oxytocin augmentation	3920	Right sided (anterior shoulder) persistent at 13 months
4	28	2	41 + 4	2 previous CS. Repeat CS in early labour (1 cm). Intrauterine septum found at CS.	3410	Right sided (anterior shoulder) persistent at 13 months
5	38	3	36 + 5	IOL for severe PET. GDM on insulin. Failure to progress at 9 cm.	4140	Left sided (posterior shoulder) persistent at 16 months
6	37	2	40 + 0	Failure to progress at 6 cm after 28 h of oxytocin. Successful ECV.	3500	Right sided (anterior shoulder)

### Failed instrumental

Four papers describe BPI after failed instrumental birth in five neonates [12, 24, 33, 34]. A 2.85 kg baby had BPI with a right wrist drop [33]. Iffy et al. assessed the relationship between diabetes, macrosomic babies, and birth injuries. Out of 240 malpractice claims involving shoulder dystocia related injuries, an infant was born after two failed attempts at mid-cavity forceps. The authors state that the circumstances were consistent with intrapartum trauma. There were also adhesions present at CS and it was a difficult extraction. Weight was not documented [34]. Another CS was performed after failed mid-cavity forceps [24].

### Emergency caesarean section

In addition to the emergency deliveries already mentioned, a further 13 papers identified 65 babies with BPI who were born by emergency CS for either maternal or fetal concerns.

A woman was diagnosed with a uterine fibroma at approximately 8 weeks’ gestation. At the 18–20 week scan, persistent asymmetry in arm position was noted in the fetus, and decreased movements of the right arm. Emergency CS, described as uncomplicated, was performed 7 h after labour due to presumed fetal distress. The baby weighed 3.56 kg and sustained a BPI. Gestation was not documented. The asymmetry noted at scan, and muscle atrophy at birth, supported compression of the brachial plexus by the fibroma, or uterine maladaptation. However, a stretch injury to the brachial plexus was supported by electromyogram findings. The authors concluded that the injury was likely due to two mechanisms; long standing uterine compression, and a stretch injury to the already weakened brachial plexus [35]. Sinclair et al. describe a case of combined intrauterine vascular insufficiency and complete BPI in an infant born by CS. A 27 year-old primiparous woman underwent emergency CS at 32 weeks’ gestation for hypertension and concerns regarding reduced fetal movements on scan. A 1.58 kg baby was born with Apgars of 5 and 9 at 1 and 5 min respectively. Its right arm became necrotic and required a below elbow amputation at 3 weeks of age. The baby was also diagnosed with C5-T1 brachial plexus palsy and made a complete recovery by 12 weeks of age. The authors concluded that the situation demonstrated a different mechanism of brachial plexus palsy, as they claimed that traction at delivery was eliminated, and long-standing stretch of the brachial plexus could have led to the BPI. This is a rare exception with clear evidence of a likely intrauterine contributing factor to BPI, as a result of a complex condition [41].

Seven babies diagnosed with BPI were delivered for presumed fetal distress. Two studies which identified neonatal BPI using ICD codes, described two babies with ‘abnormal’ and ‘non-reassuring’ heart rates; one baby was delivered due to preterm rupture of membranes [23], and the other was 3125 g and born at 38 weeks’ gestation [37]; the procedure was uneventful, but there was weakness of the upper limb at delivery of the latter baby [37]. A further four papers described four babies delivered via CS due to ‘fetal distress’ [24, 28, 39, 42]. Al-Qattan et al. describe a case of Klumpke’s palsy in a baby born at 35 weeks’ due to preterm labour and fetal distress. The mother had a normal BMI and the baby weighed 2.8kg. It was also diagnosed with Horner syndrome. Both injuries persisted at 4 months and required surgical correction [36].

Two babies were born by emergency CS due to maternal concerns. A 38 year-old woman with placenta praevia presented at 34 weeks’ gestation with heavy bleeding. The baby weighed 2765 g and was extracted by his legs [37]. Another woman was delivered for toxaemia [42]. Rehm et al. and Haley et al. reported on a further 54 babies diagnosed with BPI after emergency CS [38, 40].

### Zavanelli manoeuvre

Seven papers identified 9 cases of BPI after the Zavanelli manoeuvre (cephalic replacement following intractable shoulder dystocia, and delivery by CS [54]). They are summarised in Table 3.

**Table 3:** Erb’s palsy cases after Zavanelli manoeuvre

Author	Erb’s palsy cases after Zavanelli	Details
Doty et al. [48]	1	• Shoulder dystocia required over 3 maneuvers.
Gherman et al. [49]	1	• 40 weeks’. • Diet-controlled gestational diabetes, good glycaemic control • 37 pound weight gain. • Ventouse delivery • Posterior arm impacted. • Zavanelli’s performed - ‘unsuspected macrosomic’ infant weighing 4215 g delivered 35 min after initial recognition of the shoulder dystocia. • C5-C7 brachial plexus palsy, persistent at 3 years old.
Iffy et al. [22]	1	• 39 weeks’. • High BMI. • Oxytocin augmentation. • 100-minute second stage. • Forceps delivery after failed ventouse (maternal exhaustion at + 3 to the maternal spines) • 4.4 kg baby born within 13 min by Zavanelli’s. • Left permanent brachial plexus injury.
Iffy et al. [50]	2	• Two occurred after SVD • Both cases of Erb’s palsy lasted over 6 months.
Kenaan et al. [51]	2	Case 1 • 39 weeks’. • Type 2 diabetes, BMI 35. 11 kg weight gain. • Spontaneous vaginal delivery. • Head replaced after 2 min of maneuvers. • Uncomplicated CS of 4997 g neonate. • Discharged on day 9 with resolved Erb’s. Case 2 • 39 weeks’. • BMI 29, 16 kg weight gain. • Spontaneous labor • 3 h second stage, ventouse delivery. • Zavanelli manoeuvre performed after 4 min. • Neonate weighed 4043 g. • Discharged on day 5 with resolved Erb’s palsy.
Sandmire [52]	1	• Maternal weight 206 pounds (93.4 kg). • Head replaced without difficulty • 5100 g baby delivered under general anaesthesia 12 min after head replacement. • Mild weakness of one arm.
Turrentine et al. [53]	1	• No further details other than Zavanelli manoeuvre performed. • Persisted at 8 months.

### Macrosomia and raised maternal BMI

Several babies diagnosed with BPI after CS were macrosomic (birthweight over 4 kg [55]). Torki et al. describe a 4940 g baby at 39 + 1 weeks’ gestation born to a multiparous woman by elective CS. It had severe brachial plexus palsy which persisted at one month old [46]. In a large study exploring birth weight as a predictor of BPI, two babies were diagnosed with BPI after CS; one weighed between 4 and 4.5 kg, and the other was less than 4 kg but born to a woman with diabetes (associated with increased bisacromial diameter regardless of the absolute weight). The incidence of BPI increased as neonatal weight increased [45]. A term 4.3 kg baby was born to a woman with gestational diabetes for failure to progress and fetal distress. There was no documented difficulty in performing the CS, but the baby was diagnosed with BPI. It made a full recovery [44]. Similarly, a 4280 g baby was diagnosed with BPI after being born by elective CS with no documented complications; however, the woman had had 3 previous CS and total of 9 deliveries. Of note, her previous babies weighed between 3755 and 4550 g with no reported previous BPI [37]. Aberg et al. identified 18 babies with BPI after CS; six weighed between 4 and 5 kg. They concluded that neonates of high birth weight were at higher risk of birth-related complications [43]. Similarly, a study by Sibinski et al. describes two elective sections; one for previous CS, and one for maternal request, where the average birth weight was 4.2 kg [26].

We have described cases of neonatal BPI in women with high BMIs in previous Sects.  [22, 31, 51, 52]. Eken et al. also documented a further four babies with BPI born to obese mothers [47].

### Elective caesarean section

Four papers describe another 22 infants with BPI diagnosed following elective CS [13, 28, 38, 42] beyond those described in previous sections. Alexander et al. used ICD-9 codes to identify fetal injury associated with caesarean delivery. Four babies sustained BPI after elective CS [28]. The circumstances of the delivery were not described. In another study, an infant born by elective CS was noted to have a hyperflexed left wrist on ultrasound. At birth, the left arm was smaller than the right with flaccid paresis. No cause was found [13].

## Discussion

### Main findings

This study identified 299 infants who sustained BPI after CS, with risk factors present in over 50% of cases. Most papers in this systematic review have identified factors associated with a technically difficult/traumatic delivery. 33 babies sustained BPI after CS for malpresentation, 36 after CS for obstructed labour, 5 after CS for failed instrumental delivery, 9 after Zavanelli’s manoeuvre, 68 after emergency CS, 6 after CS with background of previous surgery, and 24 babies sustained BPI after CS in the context of fetal macrosomia and/or raised maternal BMI. 7 preterm babies with low birth weight sustained BPIs after CS. There were 3 rare cases of babies with complex medical conditions and a likely physical vulnerability to injury.

### Strengths and limitations

To our knowledge, this is the first systematic review to explore the risk factors for BPI after CS. There were no language restrictions. The majority of studies were case series without control groups. A number of studies were retrospective and relied on ICD codes, so the incidence of BPI after CS may have been under reported. Additionally, the proportion of cases with risk factors for difficult delivery were likely underestimated. In the paper with the largest number of BPIs after CS, Haley et al. grouped both emergency and elective CS together (n = 154), and could only report with assurance 50 cases of BPI after emergency CS as they relied on ICD codes [38]. 29 studies with no further clinical details were excluded.

### Interpretation

Authors have claimed that cases of BPI after CS are non-iatrogenic. Gherman et al. described cases of BPI after ‘atraumatic’ CS [16]. However, when one looks at the cases described in detail, undue traction cannot be safely excluded. In describing a case of BPI after elective CS for malpresentation, where the baby also sustained a clavicular fracture during difficult surgery, Iffy et al. argue that ‘the occurrence of brachial plexus injury as a result of strong traction applied during Cesarean section is by no means inconceivable’ [22]. In our opinion, a critical look at the cases reported shows that BPIs after CS are more likely to occur after considerable handling/manipulation of the fetus prior to, or at, delivery (such as in Zavanelli’s, malpresentation, obstructed labor, and macrosomia), where there is an urgent need to deliver the baby and surgical technique may be compromised by clinical urgency, and/or poor surgical access due to high maternal BMI or adhesions. This finding may not be surprising as a degree of traction is necessary to deliver a baby during CS and therefore an analogy with the mechanism of injury during vaginal delivery is plausible. Many accoucheurs may not be aware of the residual small risk of BPI during CS; cautious traction would be advisable as well as other techniques for releasing the shoulder(s), such as sliding a finger under the fetal armpit to gently deliver the baby, thereby reducing lateral traction on the head, or extending the uterine incision.

## Conclusion

Only approximately 1% of all BPI cases occur after CS, often emergency [36]. Aside from a few cases with documented concerns regarding limb abnormalities seen in antenatal scans, it is difficult to justify that the remaining cases could be due to antepartum events alone. A caveat is that looking at fetal arms and hands in the third trimester is not routine practice and even if it is, the views are usually limited by fetal position. Antenatal insults leading to BPI may be under recognised. The aetiology of BPI after CS could be multifactorial in a few cases, and the result of a combination of in-utero events and difficult extraction [13, 35, 41]. We therefore suggest that future research focusses upon the mechanism of BPI during CS, and on training junior obstetricians on safe delivery during CS, as previous practical courses such as PROMPT have highlighted that adequate training can reduce BPI in the context of vaginal birth [12, 17, 18]. It is important to recognise those that are at increased risk, and to limit lateral traction.

Although the incidence of BPI after CS is low, the presence of risk factors associated with difficult delivery should alert the accoucheurs to the risk of BPI if there is any undue traction on the baby. The risk is lower compared to a vaginal birth complicated by shoulder dystocia, but it is not zero. This systematic review casts doubt on theories seeking to use BPI after CS as evidence for attributing a large proportion of BPIs to the intrauterine environment and not the accoucheurs.

## Electronic supplementary material

Below is the link to the electronic supplementary material.

**Additional file 1**

## Data Availability

The datasets supporting the conclusions of this article are included within the article and its additional files.

## Declarations

### Human data

Each study included in the systematic review had relevant ethics approval and followed the relevant guidelines/regulations.

## References

[1] Leinberry CF, Wehbé MA (2004). Brachial plexus anatomy. Hand Clin.

[2] Andersen J, Watt J, Olson J, Van Aerde J (2006). Perinatal brachial plexus palsy. Paediatr Child Health.

[3] Thatte M, Mehta R (2011). Obstetric brachial plexus injury. Indian J Plast Surg.

[4] Narakas AO. Injuries of the brachial plexus and neighboring peripheral nerves in vertebral fractures and other trauma of the cervical spine. Der Orthopade. 2014;16(1). 3574946

[5] Chauhan SP, Chang KW-C, Ankumah N-AE, Yang LJ-S (2016). Neonatal brachial plexus palsy: obstetric factors associated with litigation. J Maternal-Fetal Neonatal Med.

[6] Yau CWH, Pizzo E, Prajapati C, Draycott T, Lenguerrand E (2018). Obstetric brachial plexus injuries (OBPIs): health-related quality of life in affected adults and parents. Health Qual Life Outcomes.

[7] Sakellariou VI, Badilas NK, Stavropoulos NA (2014). Treatment Options for Brachial Plexus Injuries. ISRN Orthop.

[8] Freeman MD, Goodyear SM, Leith WM (2016). A multistate population-based analysis of linked maternal and neonatal discharge records to identify risk factors for neonatal brachial plexus injury. Int J Gynecol Obstet.

[9] The Lancet (2018). Stemming the global caesarean section epidemic. The Lancet.

[10] Walsh JM, Kandamany N, Ni Shuibhne N, Power H, Murphy JF, O’Herlihy C (2011). Neonatal brachial plexus injury: comparison of incidence and antecedents between 2 decades. Am J Obstet Gynecol.

[11] Louden E, Marcotte M, Mehlman C, Lippert W, Huang B, Paulson A (2018). Risk factors for Brachial Plexus Birth Injury. Children.

[12] Crofts J, Lenguerrand E, Bentham G (2015). Prevention of brachial plexus injury-12 years of shoulder dystocia training: an interrupted time-series study. BJOG: An International Journal of Obstetrics & Gynaecology.

[13] Evans-Jones G (2003). Congenital brachial palsy: incidence, causes, and outcome in the United Kingdom and Republic of Ireland. Archives of Disease in Childhood - Fetal and Neonatal Edition.

[14] Graham EM, Forouzan I, Morgan MA (1997). A retrospective analysis of Erb’s palsy cases and their relation to Birth Weight and Trauma at Delivery. J Maternal-Fetal Neonatal Med.

[15] Doumouchtsis SK, Arulkumaran S (2009). Are all brachial plexus injuries caused by shoulder dystocia?. Obstet Gynecol Surv.

[16] Gherman RB, Goodwin MT, Ouzounian JG, Miller DA, Paul RH (1997). Brachial plexus palsy associated with cesarean section: an in utero injury?. Am J Obstet Gynecol.

[17] Weiner CP, Collins L, Bentley S, Dong Y, Satterwhite CL (2015). Multi-professional training for obstetric emergencies in a US hospital over a 7-year interval: an observational study. J Perinatol.

[18] Grunebaum A, Chervenak F, Skupski D (2011). Effect of a comprehensive obstetric patient safety program on compensation payments and sentinel events. Am J Obstet Gynecol.

[19] Backe B, Magnussen EB, Johansen OJ, Sellaeg G, Russwurm H (2008). Obstetric brachial plexus palsy: a birth injury not explained by the known risk factors. Acta Obstet Gynecol Scand.

[20] Donnelly V, Foran A, Murphy J, McParland P, Keane D, O’Herlihy C (2002). Neonatal brachial plexus palsy: an unpredictable injury. Am J Obstet Gynecol.

[21] Gurewitsch ED, Johnson E, Hamzehzadeh S, Allen RH (2006). Risk factors for brachial plexus injury with and without shoulder dystocia. Am J Obstet Gynecol.

[22] Iffy L, Pantages P (2005). Erb’s palsy after delivery by cesarean section. (a medico-legal key to a vexing problem). Med Law.

[23] Johnson GJ, Denning S, Clark SL, Davidson C (2020). Pathophysiologic Origins of Brachial Plexus Injury. Obstet Gynecol.

[24] McFarland LV, Raskin M, Daling JR, Benedetti TJ (1986). Erb/Duchenne’s Palsy: a consequence of fetal macrosomia and method of delivery. Obstet Gynecol.

[25] Sherman D, Halamish-Shani T, Gershtansky Y, Tilburg YT, Feingold M (2010). Analysis of Brachial Plexus Injuries reported to MRM. Harefuah.

[26] Sibiński M, Synder M (2007). Obstetric brachial plexus palsy–risk factors and predictors. Ortop Traumatol Rehabil.

[27] Wolf H, Hoeksma AF, Oei SL, Bleker OP. Obstetric brachial plexus injury: risk factors related to recovery. European Journal of Obstetrics & Gynecology and Reproductive Biology. 2000 [cited 2021 Apr 6];88(2):133–8. 10.1016/s0301-2115(99)00132-3 10690670

[28] Alexander JM, Leveno KJ, Hauth J, Landon MB, Thom E, Spong CY. National Institute of Child Health and Human Development maternal-fetal Medicine Units Network. Fetal injury associated with cesarean delivery. Obstet Gynecol. 2006;108(4):885–90. 10.1097/01.AOG.0000237116.72011.f3 17012450

[29] Alsubhi FS, Althunyan AM, Curtis CG, Clarke HM (2011). Radial nerve palsy in the newborn: a case series. CMAJ.

[30] Bhat V, Ravikumara, Oumachigui A (1995). Nerve injuries due to obstetric trauma. Indian J Pediatr.

[31] Carsi MB, Clarke AM, Clarke NP (2014). Transient neonatal radial nerve palsy. A case series and review of the literature. J Hand Ther.

[32] Ogbemudia AO, Ogbemudia EJ. Emergency caesarean delivery in prolonged obstructed labour as risk factor for obstetric fractures–a case series. Afr J Reprod Health. 2012;16(3):119–22. 23437505

[33] Malik S (2014). Traumatic peripheral neuropraxias in neonates: a Case Series. J Clin Diagn Res.

[34] Iffy L, Djordjevic MM, Apuzzio JJ, Martin JD, Sama JC (2003). Diabetes, hypertension and birth injuries: a complex interrelationship. Med Law.

[35] Alfonso I, Papazian O, Shuhaiber H, Yaylali I, Grossman JA. Intrauterine shoulder weakness and obstetric brachial plexus palsy. Pediatr Neurol. 2004;31(3):225–7. 10.1016/j.pediatrneurol.2004.02.010 15351026

[36] Al-Qattan MM, El-Sayed AAF (2016). A case of Klumpke’s obstetric brachial plexus palsy following a cesarean section. Clin Case Rep.

[37] Fogel I, Katz A, Sela HY, Lebel E. Brachial plexus birth palsy: incidence, natural-course, and prognostic factors during the first year of life. J Perinatol. 2021;41(7):1590–4. 10.1038/s41372-021-00972-4 33790402

[38] Haley FM, Augustine MD, Coroneos CJ, Christakis MK, Pizzuto K, Bain JR (2018). Brachial Plexus Injury in Elective Versus Emergent caesarean section: a Cohort Study. JOGC.

[39] Ouzounian J, Korst L, Miller D, Lee R (2013). Brachial plexus Palsy and Shoulder Dystocia: obstetric risk factors remain elusive. Am J Perinatol.

[40] Rehm A, Promod P, Ogilvy-Stuart A (2019). Obstetric neonatal brachial plexus and facial nerve injuries: a 17 years single tertiary maternity hospital experience. Eur J Obstet Gynecol Reproductive Biology.

[41] Sinclair C, Murray PM, Terkonda SP (2008). Combined intrauterine vascular insufficiency and Brachial Plexus Palsy: a Case Report. HAND.

[42] Wang KK, Waters PM, Bas MA, Bauer AS. Brachial Plexus Birth Injury in the Preterm Infant: suspecting the unsuspected. J Pediatr Orthop. 2020;40(9):515–9. 10.1097/BPO.0000000000001562 32271315

[43] Åberg K, Norman M, Pettersson K, Ekéus C (2016). Vacuum extraction in fetal macrosomia and risk of neonatal complications: a population-based cohort study. Acta Obstet Gynecol Scand.

[44] Al-Qattan MM, El-Sayed AAF, Al-Kharfy TM, Al-Jurayyan NAM (1996). Obstetrical Brachial Plexus Injury in Newborn Babies delivered by caesarean section. J Hand Surg.

[45] Ecker J (1997). Birth Weight as a predictor of Brachial Plexus Injury. Obstet Gynecol.

[46] Torki M, Barton L, Miller DA, Ouzounian JG (2012). Severe Brachial Plexus Palsy in Women without Shoulder Dystocia. Obstet Gynecol.

[47] Eken M, Çınar M, Şenol T, Özkaya E, Karateke A (2015). Six-year incidence and some features of cases of brachial plexus injury in a tertiary referral center. J Turkish Soc Obstetric Gynecol.

[48] Doty MS, Chauhan SP, Chang KW-C (2020). Persistence and extent of neonatal Brachial Plexus Palsy: Association with Number of Maneuvers and Duration of Shoulder Dystocia. Am J Perinatol Rep.

[49] Gherman R, Ouzounian J, Chauhan S (2010). Posterior arm shoulder Dystocia alleviated by the Zavanelli Maneuver. Am J Perinatol.

[50] Iffy L, Brimacombe M, Apuzzio JJ, Varadi V, Portuondo N, Nagy B (2007). The risk of shoulder dystocia related permanent fetal injury in relation to birth weight. Eur J Obstet Gynecol Reproductive Biology.

[51] Kenaan J, González-Quintero VH, Gilles J (2009). The Zavanelli maneuver in two cases of shoulder dystocia. J Maternal-Fetal Neonatal Med.

[52] Sandmire HF (2003). Catastrophic shoulder dystocia. Int J Gynecol Obstet [Internet].

[53] Turrentine M (1999). Adverse perinatal events and subsequent cesarean rate. Obstet Gynecol.

[54] Dharmasena D, Berg L, Hay A, Yoong W. The Zavanelli manoeuvre revisited: a review of the literature and a guide to performing cephalic replacement for severe shoulder dystocia. Eur J Obstet Gynecol Reprod Biol. 2021;266:63–73. 10.1016/j.ejogrb.2021.09.011. 10.1016/j.ejogrb.2021.09.011 34592651

[55] Said AS, Manji KP. Risk factors and outcomes of fetal macrosomia in a tertiary centre in Tanzania: a case-control study. BMC Pregnancy Childbirth 2016;16(1):243. 10.1186/s12884-016-1044-3. 10.1186/s12884-016-1044-3 PMC4997651 27557930

